# Post COVID-19, still wear a face mask? Self-perceived facial attractiveness reduces mask-wearing intention

**DOI:** 10.3389/fpsyg.2023.1084941

**Published:** 2023-01-24

**Authors:** Seung Eun Cha, Xyle Ku, Incheol Choi

**Affiliations:** ^1^Department of Psychology, Seoul National University, Seoul, Republic of Korea; ^2^Center for Happiness Studies, Seoul National University, Seoul, Republic of Korea

**Keywords:** self-perceived attractiveness, mask-wearing intention, belief, job interview, impression motivation

## Abstract

With the emerging post-COVID era, wearing face masks has become a domain of personal choice. Then, who wants to continue wearing a mask when it is no longer mandatory? In this article, we expect and examine the role of self-perceived facial attractiveness in predicting mask-wearing intention and its mechanism across three studies (total *N* = 1,030). Studies 1 and 2 demonstrated that individuals with high (vs. low) self-perceived attractiveness were less willing to wear a mask, due to a weaker endorsement of the belief that mask-wearing enhances their perceived attractiveness (i.e., mask attractiveness belief). Study 3 further revealed that this mediational association was stronger in situations where the need to deliver a favorable impression was high (job interview context) versus low (walking a dog context). Overall, we provide a novel finding that self-perceived attractiveness has significant effects on mask-wearing intention *via* mask attractiveness belief in the post-pandemic of COVID-19. Our findings suggest that mask-wearing can shift from being a self-protection measure during the COVID-19 pandemic to a self-presentation tactic in the post-pandemic era.

## Introduction

“I can’t wait to stop wearing a mask … I can’t wait to show my full face in places again.” (Anonymous internet user)

“I like to hide my face under the mask and really dread the day when mask mandates will come to an end.” (Anonymous internet user)

With the post-COVID-19 era on the horizon, the COVID-19 mask mandate (i.e., requiring wearing a mask in public) has been lifted in many countries. As reflected in the opening quotes, however, some appear to welcome the stream of the times, while others seem to wish to continue wearing a mask. What contributes to these individual differences? Although there have been some heated discussions on the political underpinnings and individual freedom related to mask-wearing during the pandemic, mandatory mask-wearing was widely regarded as one of the most protective health behaviors during the pandemic (e.g., Latkin et al., 2021). However, as the post-COVID-19 era emerged with mask mandates being lifted, it seems that other psychological predictors might contribute to mask-wearing intention. The present study addresses this question by focusing on a psychological variable—self-perceived (facial) attractiveness. Specifically, we propose in this brief report that those with higher self-perceived attractiveness are *less* willing to wear a mask as they believe that wearing a mask hinders the opportunities to deliver a favorable impression to others.

Self-perceived attractiveness is defined as individuals’ self-concept or beliefs about their physical appearances (Feingold, 1992; Belmi and Neale, 2014). Research shows that individuals who perceive themselves as more (vs. less) attractive possess more socially desirable attributes (e.g., extroverted and sociable; Feingold, 1992), have higher self-esteem (Bale and Archer, 2013), and enjoy better mental and physical health (Borráz-León et al., 2021). Moreover, the literature suggests that there are behavioral differences between attractive and unattractive individuals (for a review, see Langlois et al., 2000). For example, individuals higher in self-perceived attractiveness are more likely to enact self-interested behavior (Teng et al., 2022) and less likely to donate to a social equality movement (Belmi and Neale, 2014). Hence, prior research suggests that the degree of self-perceived attractiveness is associated with a host of social behaviors. Reflecting on these relationships, we theorize that one’s self-perceived attractiveness can relate to mask-wearing intention as mask-wearing during the COVID-19 pandemic has received significant attention from individuals.

In essence, a mask covers the lower half of the face. As essential cues that signal (un) attractiveness (e.g., facial symmetry; see Little et al., 2011) can be censored with a mask, mask-wearing might critically influence how one’s attractiveness is perceived. In support of this tenet, prior research demonstrated that their baseline facial attractiveness largely determines how the attractiveness of individuals’ mask-worn faces is perceived. Specifically, relatively unattractive individuals are deemed more attractive with masks (Patel et al., 2020; Pazhoohi and Kingstone, 2022), whereas relatively attractive individuals’ mask-worn faces are perceived as less attractive (Kamatani et al., 2021). Overall, previous findings suggest that mask-wearing enhances perceived attractiveness among unattractive individuals, while the opposite is true for attractive individuals.

Individuals may also be highly aware of the mask-attractiveness relationship during the pandemic. For example, Koreans coined the term “ma-gi-kkun,” literally referring to less attractive people who intentionally wear a mask to deliver a more favorable impression than their uncovered face would do. Additionally, similar terms are used in America (i.e., mask-fisher) and Japan (i.e., masuku-sagi) to indicate how relatively unattractive individuals appear more attractive when using a mask to cover up. The use of these neologisms suggests that individuals who perceive themselves as attractive (vs. unattractive) are more (vs. less) likely to believe that wearing masks gets in the way of gaining more favorable judgments from others regarding their attractiveness.

Taken together, we expect that individuals who perceive themselves as attractive (unattractive) will be less (more) likely to endorse the belief that wearing a mask *enhances* their perceived attractiveness (henceforth called *“mask attractiveness belief”*). We further expect that this belief, in turn, will lead to a lower (higher) intention to wear a mask. To test our hypothesis, we focus mainly on a job interview context where interviewees’ physical appearance considerably affects their interview outcomes (Langlois et al., 2000; Barrick et al., 2009; Tu et al., 2022).

## Overview of the present study

**Figure 1:**
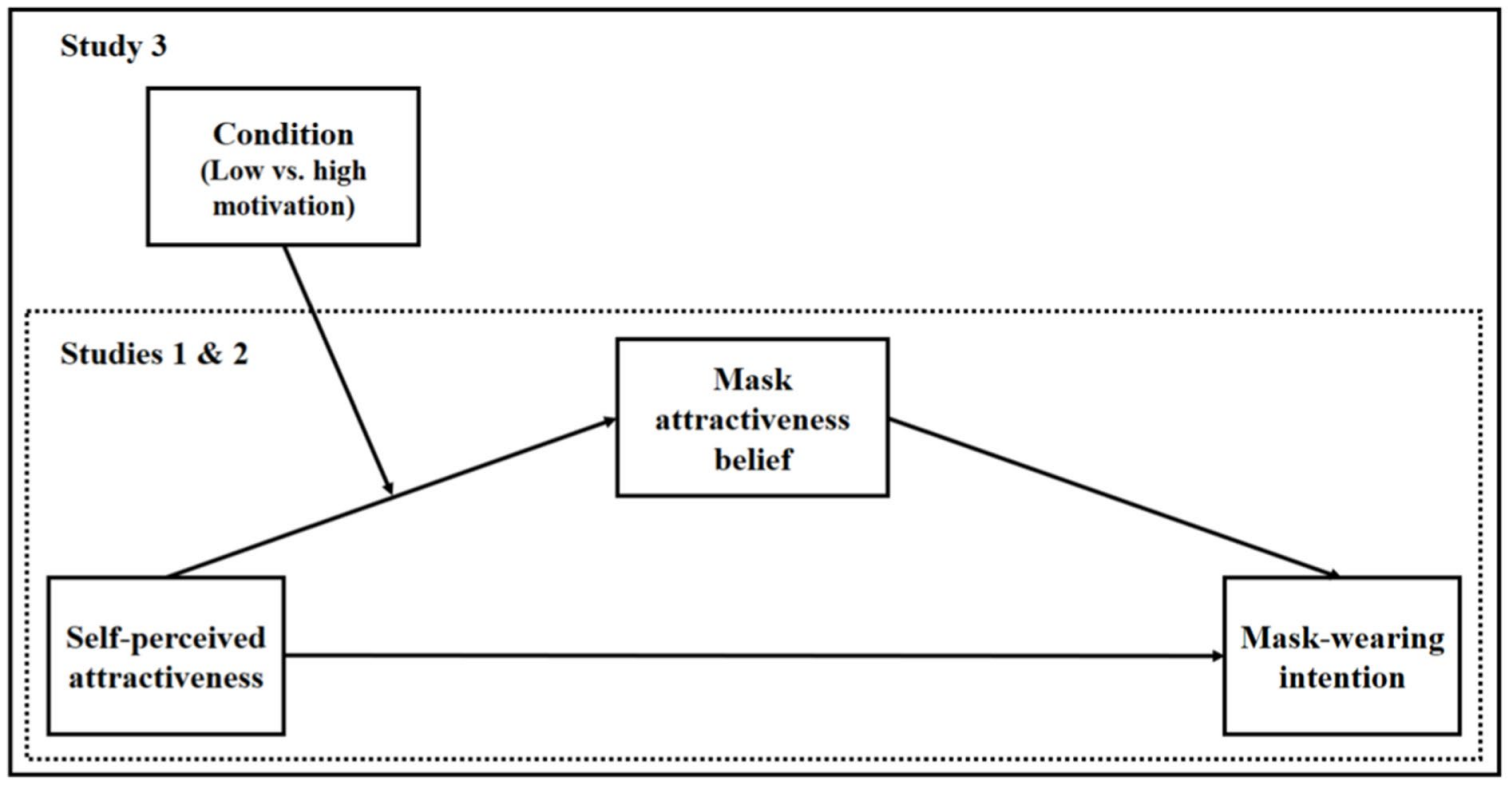
Conceptual framework. Mask attractiveness belief = belief that wearing a face mask enhances perceived attractiveness.

Across three studies (see Figure 1), we examine our hypothesis that mask attractiveness belief will mediate the relationship between self-perceived attractiveness and mask-wearing intention. In Study 1, we provide initial evidence supporting our prediction. We then replicate and extend our hypothesized model in Study 2 while controlling for other possible mediators in our model. In Study 3, we probe the boundary condition of our hypothesized mediation by comparing situations that elicit low vs. high impression motivation.

## Statistical analyses

We report all descriptive statistics, correlations, and results for the impacts of demographics on our focal variables across Studies 1 to 3 in the Supplemental Material. In Studies 1 and 2, we used a bootstrap mediation analysis by utilizing SPSS PROCESS macro Model 4 (Hayes, 2013) to examine simple and multiple mediations, respectively. In Study 3, we used a first-stage moderated mediation model to test whether our mediational model is moderated by the level of impression motivation (Edwards and Lambert, 2007), using SPSS PROCESS macro Model 7 (Hayes, 2013). We applied 10,000 bootstrap sampling with 95% bias-corrected confidence intervals for testing our hypothesized mediation effects throughout Studies 1 to 3. We note that bootstrapping procedure for mediational analyses is a robust statistical analysis that does not impose a normality assumption (Preacher and Hayes, 2008).

## Study 1

### Sample and procedure

We recruited 244 participants from Amazon Mechanical Turk (Mturk), from whom we obtained online voluntary informed consent before the study onset. Individuals residing in the United States were eligible to participate in all studies reported in this article. On average, participants were 33.03 years old (SD = 7.30) and 46.7% male. Participants self-identified as White (73.4%), Black or African American (9%), Asian or Asian American (8.2%), or others (9.4%).^1^

### Measures

Unless otherwise indicated, participants responded on a scale anchored at 1 (*strongly disagree*) and 7 (*strongly agree*) for all scales used throughout Studies 1 to 3.

#### Self-perceived attractiveness

We measured self-perceived attractiveness using a 3-item scale from Belmi and Neale (2014) (Cronbach’s *α* = 0.96; e.g., “*I think I have a lot of attractive qualities*.”). Participants were specifically asked to self-evaluate their *facial* appearance.

#### Mask attractiveness belief

Mask attractiveness belief was assessed using a single item: *“Do you think the interviewers will perceive you as more attractive with a face mask?”*

#### Mask-wearing intention

Participants were asked to imagine a situation where they received an email regarding a job interview invitation from a company they applied to. After fully imagining the situation, they indicated their mask-wearing intention on a single item (“*If wearing a face mask is optional in this interview session, would you wear a face mask during the company interview?”*) on a scale of 1 (*absolutely no*) to 10 (*absolutely yes*).

#### Covariates

We finally measured the possible correlates of mask-wearing intention—COVID-19 fear (Chen and Lei, 2021), self-esteem (Leary, 2004), and demographic variables including age, gender, and ethnicity (Hitt and Barr, 1989; Reece, 2022). Participants rated how much fear they felt toward COVID-19 using 7 items from Ahorsu et al. (2020) (*α* = 0.92; e.g., *“I am most afraid of coronavirus-19.”*) on a 5-point scale. Participants reported their self-esteem using the 10-item Rosenberg’s (1965) Self-Esteem scale (*α* = 0.93; e.g., “*On the whole, I am satisfied with myself.”*) on a 4-point scale. Finally, participants indicated their age, gender (0 = *male*, 1 = *female*), and ethnicity (0 = *White*, 1 = *ethnic minority*).^2^

### Results

Self-perceived attractiveness was negatively correlated with mask attractiveness belief (*r* = −0.26, *p* < 0.001) and mask-wearing intention (*r* = −0.17, *p* = 0.009), both of which were positively correlated with each other (*r* = 0.49, *p* < 0.001).

We then tested our main mediational hypothesis while accounting for the relevant control variables specified above (see Table 1). In support of our hypothesis, mask attractiveness belief mediated the link between self-perceived attractiveness and mask-wearing intention (*B* = −0.20, SE = 0.08, 95% CI [−0.364, −0.054]). Specifically, self-perceived attractiveness was negatively associated with mask attractiveness belief (*B* = −0.26, SE = 0.08, *p* = 0.002), which in turn, was positively associated with mask-wearing intention in a job interview (*B* = 0.75, SE = 0.12, *p* < 0.001).

**Table 1:** Regression results for simple mediation in Study 1.

Predictor	Mask attractiveness belief			Mask-wearing intention		
	*B*	SE	*t*	*B*	SE	*t*
Constant	4.38	0.69	6.39***	2.45	1.32	1.70
Self-perceived attractiveness	−0.26	0.08	−3.20**	0.04	0.15	0.24
Mask attractiveness belief				0.75	0.12	6.50***
COVID-19 fear	0.49	0.11	4.59***	0.95	0.20	4.78***
Self-esteem	−0.10	0.17	−0.58	−0.83	0.31	−2.69**
Age	−0.01	0.01	−1.07	0.03	0.02	1.37
Gender	−0.02	0.20	−0.12	0.38	0.35	1.09
Ethnicity	0.001	0.23	0.01	0.49	0.41	1.20
$R^{2}$		0.16			0.37	
	Bootstrapping effect		SE		95% CI (LL, UL)	
Total effect	−0.160		0.16		[−0.471, 0.150]	
Direct effect	0.036		0.15		[−0.257, 0.329]	
Indirect effect *via* mask attractiveness belief	−0.196		0.08		[−0.364, −0.054]	

Attractiveness belief = belief that wearing a face mask enhances perceived attractiveness. B indicates unstandardized regression coefficients, and SE indicates standard error. ***p < .001, **p < .01, *p < .05.

### Discussion

Study 1 provided initial evidence supporting the link between self-perceived attractiveness and mask-wearing intention *via* mask attractiveness belief. Specifically, individuals with higher self-perceived attractiveness were less likely to endorse the belief that mask-wearing enhances their perceived attractiveness, which further dampened their mask-wearing intention in job interviews.

Although the findings of Study 1 are informative, they provide us with preliminary evidence because a simple moderation model was examined. To demonstrate the robustness of the mediation effect of mask attractiveness belief in our model, we probe in Study 2 whether mask attractiveness belief still plays a key mediating role when controlling for other relevant mediators.

## Study 2

In Study 2, we take into account individuals’ beliefs about the impact of mask-wearing on perceived competence and trustworthiness as possible alternative mechanisms, as copious research has shown that these two characteristics significantly influence interviewer ratings (e.g., Feingold, 1992; Amaral et al., 2019). Moreover, it has been repeatedly shown that attractive individuals tend to gain more favorable evaluations regarding the two domains (Wilson and Eckel, 2006; Talamas et al., 2016). Hence, we test our hypothesis, while including control mediators—beliefs that mask-wearing enhances perceived trustworthiness and competence.

### Sample and procedure

We initially recruited 353 Mturk users. Nine participants who failed to follow the instructions were excluded from the analysis, resulting in 344 participants. On average, participants were 32.38 years old (SD = 6.19) and 45.1% male. Participants self-identified as White (73.8%), Black or African American (9.6%), Asian or Asian American (7.8%), Hispanic or Latino (6.1%), or others (2.6%).

### Measures

#### Self-perceived attractiveness

Self-perceived attractiveness was measured using the same scale from Study 1 (*α* = 0.96).

#### Presentation of job interview setting

To help participants imagine a job interview more vividly, we first instructed participants to recall a company that they desired to be employed. If they were not currently motivated to be employed, they were requested to recall a well-known company. Participants were further asked to write down the name of the company, which was later addressed as company A. They then read a hypothetical email from company A for a job interview (see Appendix).

#### Mask trustworthiness, competence, and attractiveness belief

For measuring beliefs about mask-wearing, three items were provided as indicated: *“Do you think the interviewers will perceive you as more [trustworthy/competent/attractive] with a face mask?”* For succinctness, each belief is referred to as a mask [trustworthiness/competence/attractiveness] belief. All items were randomly presented.

#### Mask-wearing intention

Participants rated their mask-wearing intention on a single item: *“Would you wear a face mask during the company A interview?”*

#### Covariates

Participants reported their gender (0 = *male*, 1 = *female*), age, ethnicity (0 = *White*, 1 = *ethnic minority*), and COVID-19 fear (*α* = 0.92; as in Study 1).

### Results

There were negative associations of self-perceived attractiveness with mask attractiveness belief (*r* = −0.18, *p* = 0.001) and mask-wearing intention (*r* = −0.09, *p* = 0.081) while attractiveness belief positively correlated with mask-wearing intention (*r* = 0.47, *p* < 0.001). Additionally, mask competence belief and mask trustworthiness belief were positively correlated with mask-wearing intention (*r* = 0.57, *p* < 0.001; *r* = 0.52, *p* < 0.001, respectively), but were not significantly correlated with self-perceived attractiveness (*r* = −0.03, *p* = 0.53; *r* = −0.01, *p* = 0.81, respectively).

As shown in Table 2, the parallel multiple mediations showed that the link between self-perceived attractiveness and mask attractiveness belief was significant (*B* = −0.20, SE = 0.06, *p* = 0.002), but was not significant for mask trustworthiness belief (*B* = 0.02, SE = 0.06, *p* = 0.74) or mask competence belief (*B* = −0.01, *SE* = 0.06, *p* = 0.84). Furthermore, mask attractiveness belief positively predicted mask-wearing intention (*B* = 0.20, SE = 0.06, *p* = 0.002), and mask competence belief also was positively associated with mask-wearing intention (*B* = 0.37, SE = 0.09, *p* < 0.001). However, mask trustworthiness belief was not related to mask-wearing intention (*B* = 0.14, SE = 0.09, *p* = 0.10). Overall, our results showed that the mediational effect of mask attractiveness belief was significant (*B* = −0.04, SE = 0.02, CI [−0.080, −0.006]), whereas showing non-significant mediational effects of mask trustworthiness belief (*B* = 0.003, SE = 0.01, CI [−0.020, 0.032]) and mask competence belief (*B* = −0.005, SE = 0.02, CI [−0.055, 0.046]).

**Table 2:** Regression results for parallel mediation in Study 2.

Predictor	Mask attractiveness belief			Mask trustworthiness belief			Mask competence belief			Mask-wearing intention		
	*B*	SE	*t*	*B*	SE	*t*	*B*	SE	*t*	*B*	SE	*t*
Constant	3.95	0.58	6.87***	4.06	0.60	6.79***	4.15	0.59	7.02***	1.05	0.61	1.73
Self-perceived attractiveness	−0.19	0.06	−3.20**	0.02	0.06	0.328	−0.01	0.06	−0.21	−0.06	0.06	−1.05
Mask attractiveness belief										0.20	0.06	3.14**
Mask trustworthiness belief										0.14	0.09	1.65
Mask competence belief										0.37	0.09	4.05***
COVID-19 fear	0.37	0.09	4.27***	0.43	0.09	4.73***	0.43	0.09	4.82***	0.46	0.09	5.25***
Age	−0.01	0.01	−0.56	−0.01	0.01	−0.40	−0.01	0.01	−0.56	0.01	0.01	0.98
Gender	−0.02	0.17	−0.12	−0.18	0.18	−1.01	−0.15	0.18	−0.86	0.01	0.17	0.04
Ethnicity	0.27	0.20	1.35	−0.26	0.20	−1.29	−0.08	0.20	−0.38	0.33	0.19	1.70
$R^{2}$		0.10			0.06			0.07			0.43	
	Bootstrapping effect				SE				95% CI (LL, UL)			
Direct effect	−0.06				0.06				[−0.125, 0.049]			
Indirect effect total	−0.04				0.04				[−0.238, 0.035]			
Indirect effect *via* attractiveness belief	−0.04				0.02				[−0.080, −0.006]			
Indirect effect *via* trustworthiness belief	0.003				0.01				[−0.020, 0.032]			
Indirect effect *via* competence belief	−0.005				0.02				[−0.055, 0.046]			

Mask trustworthiness/competence/attractiveness belief = belief that wearing a face mask enhances perceived trustworthiness/competence/attractiveness. B indicates unstandardized regression coefficients, and SE indicates standard error. ***p < .001, **p < .01, *p < .05.

### Discussion

In Study 2, we showed that mask attractiveness significantly mediated the relationship between self-perceived attractiveness and mask-wearing intention even when controlling for other alternative beliefs—namely, mask trustworthiness/competence beliefs. By identifying that the two beliefs did not associate with self-perceived attractiveness, our findings in Study 2 verified the robustness of our hypothesized mediation model.

Although the consistent findings in Studies 1 and 2 are encouraging, it is worth noting that both studies focused on a job interview where individuals are highly motivated to create a good first impression. Therefore, a critical next step would be to probe whether our previous findings are replicated in situations where impression motivation is lower than in job interviews. Study 3 thus aims to investigate whether the degree of impression motivation sets a boundary condition for our hypothesized mediation model.

## Study 3

Previous empirical evidence suggests that individuals are heightened to detect and scrutinize how others view themselves, especially when impression motivation is high (Leary and Kowalski, 1990; Leary, 2019). We thus expect that individuals with higher self-perceived attractiveness are more likely to form beliefs about masks influencing their attractiveness when they have a higher (vs. lower) impression motivation. To examine this idea, we test a first-stage moderated mediation model in Study 3 (see Figure 1), which indicates that situations differing in impression motivation moderate the impact of self-perceived attractiveness on mask attractiveness belief, which in turn, affects mask-wearing intention.

### Sample and procedure

We initially recruited 445 Mturk users. Three participants who failed to properly answer the attention check item were excluded from the current analysis, resulting in a final sample of 442 participants. On average, participants were 34 years old (SD = 7.51) and 41.4% male. Participants self-identified as White (65.8%), Black or African American (12.7%), Asian or Asian American (9.7%), Hispanic or Latino (10.0%), or others (1.8%).

#### Experimental manipulation (condition)

To construct a scenario that induces lower impression motivation compared to a job interview, we selected a scenario where individuals engage in the mundane activity of walking their dogs. If participants were not dog owners, we asked them to imagine that they participated as a one-day dog walker. Overall, participants were randomly assigned to either a low (waking a dog; *n* = 225, 41.8% male) or a high impression motivation condition (attending a job interview as in Study 1; *n* = 217, 41.0% male).

### Measures

#### Self-perceived attractiveness

Self-perceived attractiveness was measured as in previous studies (*α* = 0.96).

#### Mask-wearing intention

Participants indicated their mask-wearing intention using two items (1 = *absolutely no*; 10 = *absolutely yes*): *“If wearing a face mask is optional [while walking a dog /in this interview session], would you wear a face mask/how likely is it that you will wear a face mask [while walking a dog/during the company interview]?”* (*α* = 0.98).

#### Mask attractiveness belief

Participants reported their mask attractiveness belief on a single item: *“In this scenario, do you think others will perceive you as more attractive with a face mask?”*

#### Manipulation check

Participants indicated their degree of impression motivation by answering three items (e.g., “*How much do you want to make a good first impression on others?*”; *α* = 0.91).

### Results

Participants in the high impression motivation condition showed heightened motivation to form a good first impression (*M* = 6.32, SD = 0.83) than those in the low impression motivation condition (*M* = 4.20, SD = 1.60), *t*(440) = 17.39, *p* < 0.001, *d* = 1.66, indicating that our manipulation was successful.

**Figure 2:**
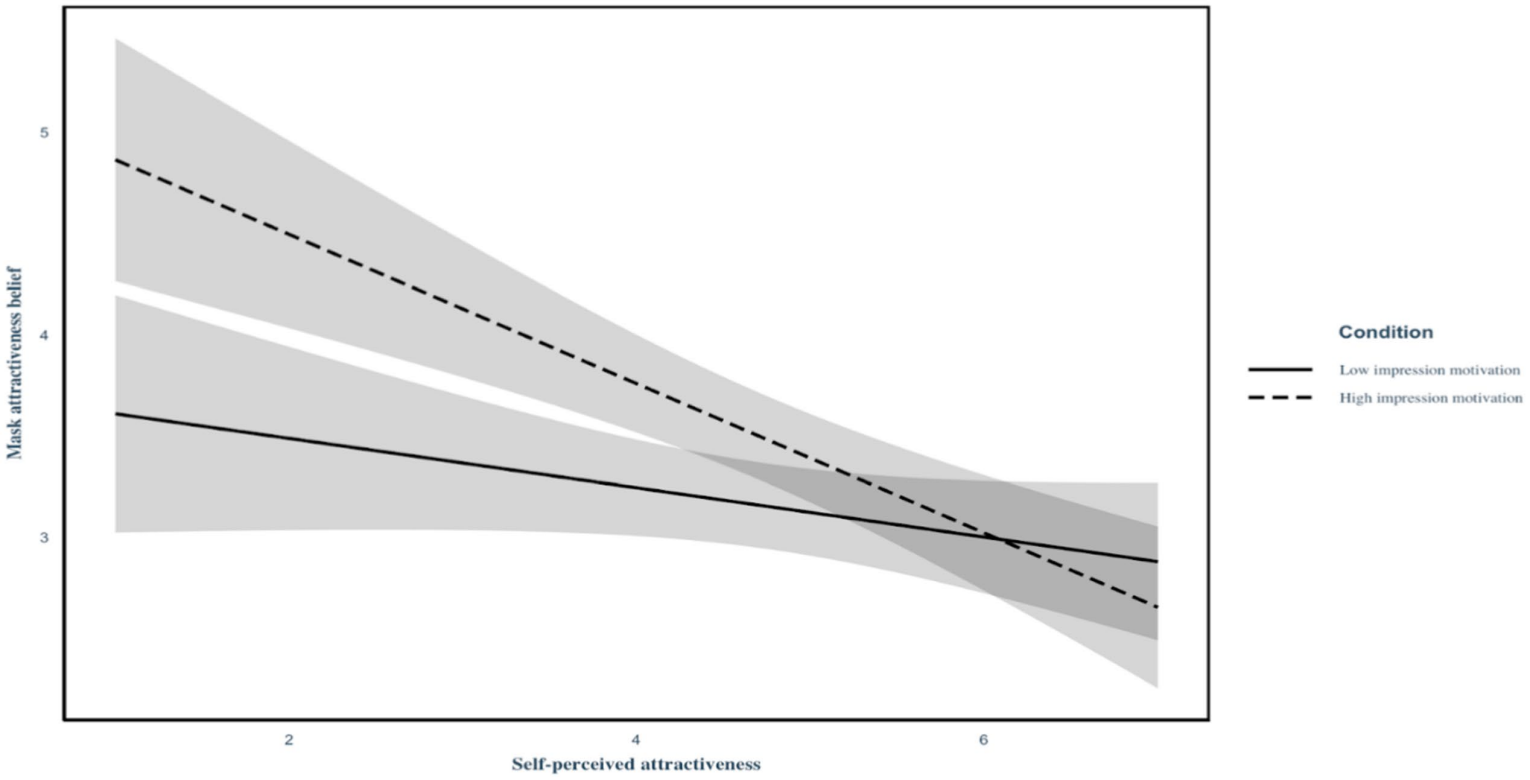
Regression slopes for the interaction of self-perceived attractiveness and the conditions on mask attractiveness belief in Study 3 (*N* = 442). Mask attractiveness belief = belief that wearing a face mask enhances perceived attractiveness. Self-perceived attractiveness was mean-centered prior to analysis.

To test our first-stage moderated mediation hypothesis, we first regressed self-perceived attractiveness, the condition (1 = *low impression motivation condition*, 2 = *high impression motivation condition*), and their interaction term on mask attractiveness belief (see Model 1 in Table 3). We found that the main effect of self-perceived attractiveness was not significant (*B* = 0.13, SE = 0.17, *p* = 0.45), whereas the main effect of the condition was significant (*B* = 0.33, SE = 0.15, *p* = 0.03). More importantly, the interaction effect was significant (*B* = −0.25, SE = 0.11, *p* = 0.02), such that self-perceived attractiveness negatively predicted mask attractiveness belief in the high impression motivation condition (*B* = −0.37, SE = 0.08, *p* < 0.001), but not in the low impression motivation condition (*B* = −0.12, SE = 0.07, *p* = 0.10; see Figure 2).

**Table 3:** Test of moderated mediation model in Study 3 (*N* = 442).

Predictor	Model 1 (DV = Mask attractiveness belief)			Model 2 (DV = Mask-wearing intention)		
	*B*	SE	*t*	*B*	SE	*t*
Constant	2.82	0.24	11.73***	1.83	0.33	5.62***
Self-perceived attractiveness	0.13	0.17	0.75	−0.05	0.10	−0.52
Condition	0.33	0.15	2.15*			
Self-perceived attractiveness*Condition	−0.25*	0.11	−2.32*			
Mask attractiveness belief				0.97	0.09	10.99***
$R^{2}$		0.07			0.23	
	Bootstrapping effect		SE		95% CI (LL, UL)	
Direct effect	−0.05		0.10		[−0.252, 0.147]	
Index of moderated mediation	−0.24		0.11		[−0.454, −0.022]	

Mask attractiveness belief = belief that wearing a face mask enhances perceived attractiveness. Conditions are coded 1 (low motivation condition; walking a dog) and 2 (high motivation condition; job interview). Self-perceived attractiveness was mean-centered prior to analysis. *B* indicates unstandardized regression coefficients, and SE indicates standard error. ***p < .001, **p < .01, *p < .05.

Second, mask attractiveness belief, in turn, was positively associated with mask-wearing intention (*B* = 0.97, SE = 0.09, *p* < 0.001; Model 2 in Table 3). Therefore, the index of moderated mediation was found to be significant (*B* = −0.24, SE = 0.11, 95% CI [−0.457, −0.022]). That is, the indirect effect of self-perceived attractiveness on mask-wearing intention *via* mask attractiveness belief was *stronger* in the high impression motivation condition (*B* = −0.36, SE = 0.08, 95% CI [−0.513, −0.207]) than in the low impression motivation condition (*B* = −0.12, SE = 0.09, 95% CI [−0.301, 0.042]).

### Discussion

Study 3 corroborated our hypothesized effect as in Studies 1 and 2. To probe the boundary condition, we manipulated situations to activate low vs. high impression motivation and found evidence that the association of self-perceived attractiveness with mask-wearing intention was stronger when individuals had higher impression motivation. Therefore, those with less desire to form favorable impressions were less likely to form beliefs about how mask-wearing can associate with their facial attractiveness. The results demonstrate that how much the situation elicits impression motivation can serve as a boundary condition for the mediational effect of mask attractiveness belief on the relationship between self-perceived attractiveness with mask-wearing intention.

## General discussion

In this brief report, we hypothesized that those with higher self-perceived attractiveness would have lower mask-wearing intention as they are less likely to believe that mask-wearing enhances their attractiveness (i.e., mask attractiveness belief). In Study 1, we found initial evidence supporting our hypothesis through a simple mediation model while controlling for relevant covariates. In Study 2, we replicated and extended the results of Study 1 by ruling out other alternative mechanisms. Specifically, we found that the mediating impact of mask attractiveness belief remained significant, even when other beliefs were controlled for as parallel mediators (i.e., mask trustworthiness and competence belief). Therefore, Studies 1 and 2 underscored that individuals mirror how others view mask-worn faces of attractive individuals (Patel et al., 2020; Kamatani et al., 2021) to actively form mask attractiveness belief. In Study 3, we tested the boundary condition of our hypothesized model and found that the mediation of mask attractiveness belief was only relevant in situations where others’ judgments highly mattered (i.e., job interview) compared to where it mattered less (i.e., dog walking). Thus, our novel findings demonstrated that the impact of self-perceived attractiveness on mask-wearing intention *via* attractiveness belief is only significant in situations where one is motivated to impress.

### Theoretical and practical contributions

Our findings offer several important contributions to theory and practice. First, it is noteworthy that extant literature has focused primarily on mask-wearing intention in the context of health behaviors during the pandemic. Instead, the present study provides a fresh window into how individuals may use face masks beyond the means of health behaviors, as mask-wearing has become optional in many countries. It is worth noting that mask attractiveness belief was related to mask-wearing intention to a similar degree of COVID-19 fear in Study 1 (*r* = 0.49, *p* < 0.001 versus *r* = 0.45, *p* < 0.001) and Study 2 (*r* = 0.47, *p* < 0.001 versus *r* = 0.41, *p* < 0.001). Therefore, our results demonstrate that mask-wearing can serve two functions in the post-pandemic era—self-presentation and self-protection.

Second, our study took a novel predictor, self-perceived attractiveness, to examine the antecedents of mask-wearing intention. Although prior work suggests that mask-wearing can be used for cosmetic purposes (Miyazaki and Kawahara, 2016), to the best of our knowledge, the current work is the first to empirically demonstrate its association with self-perceived attractiveness. As facial attractiveness constitutes a variety of facial features that are not confined to the upper half of the face (for a review, see Little et al., 2011), we can interpret that attractive individuals might be less willing to wear masks in order to display their attractive facial features more. Overall, the current investigation contributes to the literature by providing novel evidence that self-perceived attractiveness influences how individuals form beliefs about themselves that lead to behavioral intentions (Belmi and Neale, 2014).

Finally, our research offers practical implications. Although attractiveness should not be a factor that dictates one’s recruitment outcomes, numerous findings point to this dim reality (Barrick et al., 2009). Our results consistently demonstrated that self-perceived unattractive individuals were more willing to wear a mask, as they believed it would benefit their attractiveness. These findings suggest that individuals are highly aware of the benefits of being physically attractive during the recruitment process, driving them to enhance their physical attractiveness. Therefore, organizational selectors need to be cognizant of and counteract attractiveness bias that is still present in the recruiting process (i.e., structuralized interview; Barrick et al., 2009).

### Limitations and future research

The present investigation has several limitations. The first concern is that we primarily focused on job interviews to investigate situations that trigger high impression motivation. However, not all situations involve one-time encounters, as in job interviews. For example, individuals might also be highly motivated to impress others when going on a blind date. In these scenarios, however, self-perceived attractiveness may not necessarily lead to higher mask-wearing intention as they anticipate multiple face-to-face interactions following their initial date. Given this, it would be interesting for future research to probe whether situations that elicit high impression motivation yet vary in their expected future interactions (e.g., one-time vs. lasting interaction) influence our hypothesized model.

Another limitation is that we cannot rule out the possibility of other psychosocial factors affecting our results. For example, upper-class individuals are perceived as more attractive (Bjornsdottir and Rule, 2017; Luoto et al., 2021). Furthermore, the facial features of the higher social class lead to higher employment suitability (Stephens et al., 2014; Bjornsdottir and Rule, 2017). Thus, participants’ social class might be confounded in our results. Regarding mask-wearing intention, mask-wearing policies have been hotly debated in the United States across the political spectrum, with those identifying as Republicans generally being against public mask-wearing (Gelfand et al., 2022). Therefore, we can assume that our hypothesized path may be weaker among politically conservative participants. Although our focus in the current research did not include socio-political variables, the potential roles of these factors deserve attention in future research.

Finally, prior research shows that individuals with higher self-perceived facial attractiveness have better physical and mental health outcomes (Feingold, 1992; Borráz-León et al., 2021), which is also reflected in how others perceive attractive individuals (Jones et al., 2001). Given that health is a positive predictor of employment status (e.g., Carlier et al., 2014), those with a higher sense of self-perceived attractiveness are likely to believe that mask-wearing prevents signaling better health, leading them to show lower mask-wearing intention. Thus, it is worth testing other possible mediators, including health-related beliefs, in future studies.

## Data availability statement

The raw data supporting the conclusions of this article will be made available by the authors, without undue reservation.

## Ethics statement

The studies involving human participants were reviewed and approved by Institutional Review Board of Seoul National University. The patients/participants provided their written informed consent to participate in this study.

## Author contributions

SC: conceptualization, methodology, formal analysis, investigation, data curation, writing–original draft, review and editing, and visualization. XK: conceptualization, methodology, formal analysis, investigation, writing–original draft, and review and editing. IC: project administration, funding acquisition, and writing–review and editing. All authors contributed to the article and approved the submitted version.

## Funding

This research was funded by the Center for Happiness Studies *via* the Center for Social Sciences at Seoul National University.

## Conflict of interest

The authors declare that the research was conducted in the absence of any commercial or financial relationships that could be construed as a potential conflict of interest.

## Publisher’s note

All claims expressed in this article are solely those of the authors and do not necessarily represent those of their affiliated organizations, or those of the publisher, the editors and the reviewers. Any product that may be evaluated in this article, or claim that may be made by its manufacturer, is not guaranteed or endorsed by the publisher.

## Footnotes

^1^Due to the ethnicity option omission for Hispanic or Latino in the current survey, they were identified as others.

^2^Considering the sample size, we dummy-coded White as 0 and others (i.e., ethnic minority) as 1 throughout Studies 1 and 2, following previous research (e.g., Belmi and Neale, 2014).
