# Supplementary material for: Post COVID-19, still wear a face mask? Self-perceived facial attractiveness reduces mask-wearing intention
Source: Front Psychol. 2023 Jan 24;14:1084941. doi: 10.3389/fpsyg.2023.1084941 (PMC9904203; doi:10.3389/fpsyg.2023.1084941)
Supplement: Supplementary file 1 [file Data_Sheet_1.docx]

**Post COVID-19, still wear a face mask?**

**Self-perceived facial attractiveness reduces mask-wearing intention**

*Frontiers in Psychology*

Online Supplemental Material

**Table S1**

*Descriptive Statistics and Correlations among the Variables in Study 1 (N = 244)*

|  | *M* (*SD*) | 1 | 2 | 3 | 4 | 5 | 6 | 7 | 8 |
| --- | --- | --- | --- | --- | --- | --- | --- | --- | --- |
| 1. Mask-wearing intention | 6.13 (3.34) | - | .000 | .009 | .000 | .000 | .644 | .099 | .011 |
| 2. Mask attractiveness belief | 3.48 (1.63) | .488 | - | .000 | .000 | .000 | .310 | .774 | .255 |
| 3. Self-perceived attractiveness | 4.64 (1.42) | $-$.167 | $-$.255 | - | .000 | .751 | .851 | .350 | .255 |
| 4. Self-esteem | 2.91 (0.69) | $-$.299 | $-$.224 | .533 | - | .002 | .479 | .247 | .674 |
| 5. COVID-19 fear | 2.21 (0.98) | .449 | .308 | $-$.020 | $-$.199 | - | .894 | .072 | .000 |
| 6. Age | 33.03 (7.30) | .030 | $-$.065 | $-$.012 | .046 | $-$.009 | - | .548 | .219 |
| 7. Gender | - | .106 | .019 | .060 | $-$.074 | .115 | $-$.039 | - | .855 |
| 8. Ethnicity | - | .163 | .073 | .073 | .027 | .290 | $-$.079 | $-$.012 | - |

*Note*. Mask attractiveness belief = belief that wearing a face mask enhances perceived attractiveness. Gender is coded as 0 (*male*) and 1 (*female*). Ethnicity is coded as 0 (*White*) and 1 (*ethnic minority*). Numbers above the diagonal are *p* values of each correlation coefficient.

**Table S2**

*Descriptive Statistics and Correlations among the Variables in Study 2 (N = 344)*

|  | *M* (*SD*) | 1 | 2 | 3 | 4 | 5 | 6 | 7 | 8 | 9 |
| --- | --- | --- | --- | --- | --- | --- | --- | --- | --- | --- |
| 1. Mask-wearing intention | 5.58 (1.93) | - | .000 | .000 | .000 | .081 | .000 | .935 | .273 | .015 |
| 2. Mask trustworthiness belief | 4.82 (1.63) | .516 | - | .000 | .000 | .814 | .000 | .580 | .868 | .819 |
| 3. Mask attractiveness belief | 3.78 (1.60) | .473 | .497 | - | .000 | .001 | .000 | .383 | .448 | .083 |
| 4. Mask competence belief | 4.77 (1.62) | .567 | .825 | .559 | - | .528 | .000 | .429 | .797 | .525 |
| 5. Self-perceived attractiveness | 4.55 (1.40) | $-$.094 | $-$.013 | $-$.175 | $-$.034 | - | .138 | .776 | .678 | .005 |
| 6. COVID-19 fear | 2.40 (1.00) | .405 | .238 | .260 | .256 | $-$.080 | - | .282 | .000 | .001 |
| 7. Age | 32.38 (6.19) | $-$.004 | $-$.030 | $-$.047 | $-$.043 | $-$.015 | $-$.058 | - | .965 | .070 |
| 8. Gender | - | .059 | .009 | .041 | .014 | $-$.022 | .218 | $-$.002 | - | .067 |
| 9. Ethnicity | - | .132 | $-$.012 | .094 | .034 | .152 | .184 | $-$.098 | $-$.099 | - |

*Note*. Mask trustworthiness/competence/attractiveness belief = belief that wearing a face mask enhances perceived trustworthiness/competence/attractiveness. Gender is coded as 0 (*male*) and 1 (*female*). Ethnicity is coded as 0 (*White*) and 1 (*ethnic minority*). Numbers above the diagonal are *p* values of each correlation coefficient.

**Table S3**

*Descriptive Statistics and Correlations among the Variables in Study 3 (N = 442)*

|  | *M* (*SD*) | 1 | 2 | 3 | 4 | 5 | 6 | 7 |
| --- | --- | --- | --- | --- | --- | --- | --- | --- |
| 1. Mask-wearing intention | 5.05 (3.41) | - | .000 | .010 | .495 | .131 | .129 | .000 |
| 2. Mask attractiveness belief | 3.31 (1.66) | .477 | - | .000 | .074 | .747 | .155 | .035 |
| 3. Self-perceived attractiveness | 4.75 (1.44) | $-$.122 | $-$.211 | - | .554 | .946 | .046 | .921 |
| 4. Age | 34.00 (7.51) | $-$.033 | $-$.085 | $-$.028 | - | .593 | .000 | .795 |
| 5. Gender | - | .072 | .015 | $-$.003 | $-$.025 | - | .614 | .871 |
| 6. Ethnicity | - | .072 | .068 | .095 | $-$.175 | $-$.024 | - | .979 |
| 7. Condition | - | .300 | .100 | $-$.005 | $-$.012 | .008 | $-$.001 | - |

*Note*. Mask attractiveness belief = belief that wearing a face mask enhances perceived attractiveness. Gender is coded as 0 (*male*) and 1 (*female*). Ethnicity is coded as 0 (*White*) and 1 (*ethnic minority*). Condition is coded as 1 (*low impression motivation condition; walking a dog*) and 2 (*high impression motivation condition; job interview*). Numbers above the diagonal are *p* values of each correlation coefficient.

**Demographic Differences for the Focal Variables**

To help readers fully understand our results, we report additional results regarding the associations of demographic factors (i.e., age, gender, and ethnicity) with our focal variables (i.e., self-perceived attractiveness, mask attractiveness belief, and mask-wearing intention). We note that gender was coded 0 (*male*) and 1 (*female*), while ethnicity was coded 0 (*White*) and 1 (*ethnic minority*).

First, as described in Tables S1, S2, and S3, age was not significantly associated with self-perceived attractiveness (Study 1: *r* = $-$.01, *p* = .85; Study 2: *r* = $-$.02, *p* = .78; Study 3: *r* = $-$.03, *p* = .55), mask attractiveness belief (Study 1: *r* = $-$.07, *p* = .31; Study 2: *r* = $-$.05, *p* = .38; Study 3: *r* = $-$.09, *p* = .07), and mask-wearing intention (Study 1: *r* = .03, *p* = .64; Study 2: *r* = $-$.004, *p* = .94; Study 3: *r* = $-$.03, *p* = .50). Second, gender was also not significantly associated with self-perceived attractiveness (Study 1: *r* = .06, *p* = .35; Study 2: *r* = .04, *p* = .45; Study 3: *r* = $-$.003, *p* = .95), mask attractiveness belief (Study 1: *r* = .02, *p* = .77; Study 2: *r* = .01, *p* = .87; Study 3: *r* = .02, *p* = .75), and mask-wearing intention (Study 1: *r* = .11, *p* = .10; Study 2: *r* = .06, *p* = .27; Study 3: *r* = .07, *p* = .13). Third, on the other hand, ethnicity showed inconsistent patterns. Ethnicity was not significantly associated with self-perceived attractiveness in Study 1 (*r* = .07, *p* = .26) and Study 2 (*r* = .09, *p* = .08), while they were significantly correlated in Study 3 (*r* = .10, *p* = .046), indicating that the scores of self-perceived attractiveness were higher among ethnic minorities than among Whites. Regarding mask attractiveness belief, ethnicity showed non-significant correlations (Study 1: *r* = .07, *p* = .26; Study 2: *r* = $-$.01, *p* = .82; Study 3: *r* = .07, *p* = .16). Finally, ethnicity was associated with mask-wearing intention in Study 1 (*r* = .16, *p* = .01) and Study 2 (*r* = .13, *p* = .02), such that ethnic minorities tended to have a stronger mask-wearing intention than Whites. However, no significant correlation was found in Study 3 (*r* = .07, *p* = .13).

Second, we further examined the associations of gender and ethnicity with our focal variables based on a one-way ANOVA, given the dichotomous nature of the variables.

**Study 1**

***Gender Difference***

There were no gender differences in self-perceived attractiveness, *F*(1, 242) = .88, *p* = .35, mask attractiveness belief, *F*(1, 242) = .08, *p* = .77, and mask-wearing intention, *F*(1, 242) = 2.74, *p* = .10 (see Table S4).

***Ethnicity Difference***

There were no ethnic differences in self-perceived attractiveness, *F*(1, 242) = 1.30, *p* = .26, and mask attractiveness belief, *F*(1, 242) = 1.30, *p* = .26. However, there was a significant difference in mask-wearing intention, *F*(1, 242) = 6.58, *p* = .01 (see Table S4), such that ethnic minorities had a stronger mask-wearing intention (*M* = 7.03, *SD* = 3.07) than Whites (*M* = 5.80, *SD* = 3.38).

**Study 2**

***Gender Difference***

There were no gender differences in self-perceived attractiveness, *F*(1, 342) = .17, *p* = .68, mask attractiveness belief, *F*(1, 342) = .58, *p* = .45, and mask-wearing intention, *F*(1, 342) = 1.20, *p* = .27 (see Table S5).

***Ethnicity Difference***

There was significant ethnic differences in self-perceived attractiveness, *F*(1, 342) = 8.13, *p* = .005, indicating that ethnic minorities perceived themselves as more attractive (*M* = 4.91, *SD* = 1.35) compared to Whites (*M* = 4.43, *SD* = 1.40). There was also significant ethnic differences in mask-wearing intention, *F*(1, 342) = 6.03, *p* = .015, such that ethnic minorities showed a stronger mask-wearing intention (*M* = 6.01, *SD* = 1.69) than Whites (*M* = 5.43, *SD* = 1.99). In addition, there was a marginally significant ethnic difference in mask attractiveness belief, *F*(1, 342) = 3.03, *p* = .08. That is, ethnic minorities tended to endorse mask-attractiveness belief more strongly (*M* = 4.03, *SD* = 1.58) than Whites (*M* = 3.69, *SD* = 1.60).

**Study 3**

In Study 3, we reported demographic differences among participants in the low impression motivation condition (see Table S6) and those in the high impression motivation condition (see Table S7), respectively.

***Gender Differences***

There were no gender differences in self-perceived attractiveness, *F*(1, 223) = .13, *p* = .72, mask attractiveness belief, *F*(1, 223) = .11, *p* = .74, and mask-wearing intention, *F*(1, 223) = .13, *p* = .72, among participants in the low impression motivation condition. Similarly, there were no gender differences in self-perceived attractiveness, *F*(1, 215) = .07, *p* = .79, and mask attractiveness belief, *F*(1, 215) = .01, *p* = .93, among those in the high impression motivation condition. Yet, we found a marginally significant gender difference in mask-wearing intention, *F*(1, 215) = 3.28, *p* = .07, such that female participants showed a stronger mask-wearing intention (*M* = 6.43, *SD* = 3.23) than male participants (*M* = 5.61, *SD* = 3.34).

***Ethnicity Differences***

Among participants in the low impression motivation condition, there were no ethnic differences in self-perceived attractiveness, *F*(1, 223) = 1.77, *p* = .19, and mask-wearing intention, *F*(1, 223) = 1.72, *p* = .19. Conversely, there was a marginally significant ethnic difference in mask attractiveness belief, *F*(1, 223) = 3.77, *p* = .054, such that ethnic minorities had mask attractiveness belief (*M* = 3.45, *SD* = 1.75) more strongly than Whites (*M* = 2.99, *SD* = 1.67). Among those in the high impression motivation condition, there were no ethnic differences in all focal variables: self-perceived attractiveness, *F*(1, 215) = 2.24, *p* = .14, mask attractiveness belief, *F*(1, 215) = .00, *p* = .99, and mask-wearing intention, *F*(1, 215) = .90, *p* = .34.

**Table S4**

*Demographic Differences in Focal Variables in Study 1 (N = 244)*

|  |  | Self-perceived attractiveness | | | Mask attractiveness belief | | | Mask-wearing intention | | |
| --- | --- | --- | --- | --- | --- | --- | --- | --- | --- | --- |
| Predictor | *n* | *M* | *SD* | *F* | *M* | *SD* | *F* | *M* | *SD* | *F* |
| Gender |  |  |  |  |  |  |  |  |  |  |
| Male | 114 | 4.55 | 1.48 | .88 | 3.45 | 1.56 | .08 | 5.75 | 3.28 | .10 |
| Female | 130 | 4.72 | 1.36 |  | 3.51 | 1.69 |  | 6.46 | 3.37 |  |
| Ethnicity |  |  |  |  |  |  |  |  |  |  |
| White | 179 | 4.58 | 1.35 | .88 | 3.41 | 1.62 | 1.30 | 5.80 | 3.38 | 6.58* |
| Ethnic minority | 65 | 4.82 | 1.58 |  | 3.68 | 1.65 |  | 7.03 | 3.07 |  |

*Note*. * *p* < .05.

**Table S5**

*Demographic Differences in Focal Variables in Study 2 (N = 344)*

|  |  | Self-perceived attractiveness | | | Mask attractiveness belief | | | Mask-wearing intention | | |
| --- | --- | --- | --- | --- | --- | --- | --- | --- | --- | --- |
| Predictor | *n* | *M* | *SD* | *F* | *M* | *SD* | *F* | *M* | *SD* | *F* |
| Gender |  |  |  |  |  |  |  |  |  |  |
| Male | 155 | 4.59 | 1.37 | .17 | 3.71 | 1.63 | .58 | 5.46 | 1.96 | 1.20 |
| Female | 189 | 4.52 | 1.44 |  | 3.84 | 1.58 |  | 5.69 | 1.91 |  |
| Ethnicity |  |  |  |  |  |  |  |  |  |  |
| White | 254 | 4.43 | 1.40 | 8.13** | 3.69 | 1.60 | 3.03 | 5.43 | 1.99 | 6.03* |
| Ethnic minority | 90 | 4.91 | 1.35 |  | 4.03 | 1.58 |  | 6.01 | 1.69 |  |

*Note*. ** *p* < .01, * *p* < .05.

**Table S6**

*Demographic Differences in Focal Variables among Participants in the Low Impression Motivation Condition in Study 3 (N = 225)*

|  |  | Self-perceived attractiveness | | | Mask attractiveness belief | | | Mask-wearing intention | | |
| --- | --- | --- | --- | --- | --- | --- | --- | --- | --- | --- |
| Predictor | *n* | *M* | *SD* | *F* | *M* | *SD* | *F* | *M* | *SD* | *F* |
| Gender |  |  |  |  |  |  |  |  |  |  |
| Male | 94 | 4.80 | 1.43 | .13 | 3.11 | 1.69 | .11 | 3.95 | 3.18 | .13 |
| Female | 131 | 4.73 | 1.47 |  | 3.18 | 1.71 |  | 4.11 | 3.27 |  |
| Ethnicity |  |  |  |  |  |  |  |  |  |  |
| White | 148 | 4.66 | 1.32 | 1.77 | 2.99 | 1.66 | 3.77 | 3.84 | 3.12 | 1.72 |
| Ethnic minority | 77 | 4.94 | 1.67 |  | 3.45 | 1.75 |  | 4.44 | 3.41 |  |

**Table S7**

*Demographic Differences in Focal Variables among Participants in the High Impression Motivation Condition in Study 3 (N = 217)*

|  |  | Self-perceived attractiveness | | | Mask attractiveness belief | | | Mask-wearing intention | | |
| --- | --- | --- | --- | --- | --- | --- | --- | --- | --- | --- |
| Predictor | *n* | *M* | *SD* | *F* | *M* | *SD* | *F* | *M* | *SD* | *F* |
| Gender |  |  |  |  |  |  |  |  |  |  |
| Male | 89 | 4.71 | 1.47 | .07 | 3.47 | 1.47 | .01 | 5.61 | 3.34 | 3.28 |
| Female | 128 | 4.77 | 1.42 |  | 3.49 | 1.69 |  | 6.43 | 3.23 |  |
| Ethnicity |  |  |  |  |  |  |  |  |  |  |
| White | 143 | 4.64 | 1.47 | 2.24 | 3.48 | 1.66 | .00 | 5.94 | 3.35 | .90 |
| Ethnic minority | 74 | 4.95 | 1.37 |  | 3.49 | 1.49 |  | 6.39 | 3.18 |  |
