# Supplementary material for: Post COVID-19, still wear a face mask? Self-perceived facial attractiveness reduces mask-wearing intention
Source: Front Psychol. 2023 Jan 24;14:1084941. doi: 10.3389/fpsyg.2023.1084941 (PMC9904203; doi:10.3389/fpsyg.2023.1084941)
Supplement: Supplementary file 2 [file Data_Sheet_2.docx]

**Appendix**

**Email Script**

Dear applicants,

Thank you for submitting your application to company A. We have thoroughly checked your resume and would like to invite you to an interview at our office next Friday. The interview will be conducted by the hiring managers along with the HR team.

The current interview will take place at the Central Building at 125 West Street next Friday at 5 p.m. The interview will take about 15 minutes and will consist of a brief self-presentation followed by questions regarding your applied position.

Due to COVID-19, the HR team has decided to make wearing a face mask optional during the interview.

If you have any questions or difficulties regarding the interview, feel free to contact the HR team at hrteam2022@companyA.com.

Best,

HR team

Company A
